# Residential moves, neighbourhood walkability, and physical activity: a longitudinal pilot study in Ontario Canada

**DOI:** 10.1186/s12889-018-5858-y

**Published:** 2018-07-28

**Authors:** Patricia A. Collins, Justin Tait, Allan Fein, James R. Dunn

**Affiliations:** 1 School of Kinesiology and Health Studies, Queen’s University, Kingston, ON Canada; 2 Psychiatry and Behavioural Neurosciences, McMaster University, Hamilton, ON Canada; 3 Department of Health, Aging & Society, McMaster University, Hamilton, ON Canada

**Keywords:** Walkability, Built environment, Walk score®, Physical activity, Walking, Self-efficacy, Pilot study, Longitudinal study, Ontario

## Abstract

**Background:**

Numerous cross-sectional studies have consistently demonstrated an association between attributes of urban form or ‘walkability’ and individual- and population-level physical activity (PA) patterns. However, in the absence of longitudinal research, the self-selection problem undermines the claim that a walkable built form produces more physically active people. Through a longitudinal pilot study of ‘imminent movers’ in Ontario using a quasi-experimental approach, we sought to examine the feasibility of longitudinal methods that would produce stronger evidence for a causal relationship between the built environment and PA levels.

**Methods:**

Participants were recruited using publicly available real estate listings. Successful recruits were sent a PA diary to track their activity for a week, and were also scheduled for a 45-min phone interview that collected demographic details, neighbourhood perceptions and self-efficacy for walking, and verified the PA diary. Following their move, participants were given the same tasks and then sorted into groups based on changes in their neighbourhood walkability (measured with Walk Score) from baseline to follow-up.

**Results:**

There were challenges in recruiting a sufficient number of participants and counter-factuals to examine the relationship between changes in walkability and PA. Our limited sample showed a substantial decrease in Walk Score over the entire sample, from an average of 45.8 to 30.6, with most participants moving to less walkable areas. From baseline to follow-up, the largest declines in reported self-efficacy for walking were to grocery stores, banks, and for entertainment. For the entire sample, utilitarian PA decreased, while recreational and job-related PA increased.

**Conclusions:**

This pilot study highlighted the methodological challenges involved in collecting quasi-experimental evidence on the effect of walkable environments on PA. Additionally, the low sample size and the tendency for most participants to move to less walkable areas meant there were insufficient counter-factuals for study of the effect of walkability on PA. Despite these challenges, we saw important changes in self-efficacy for walking that were commensurate with changes to the built environment. In sum, while longitudinal research on health and the built environment is urgently needed, recruiting an adequate sample size for a quasi-experimental study such as this is extremely challenging.

## Background

Accelerating rates of obesity in Canada [1] and the US [2, 3] has led to a growing interest among scholars and public health practitioners in understanding the environmental determinants of obesity [4]. Studies of built environment attributes and physical activity have produced a number of positive relationships between urban form and physical activity [4–9], while others have documented how urban form discourages physical activity [4, 10, 11]. So-called ‘walkable’ communities are those that have high population density and diverse land uses, with amenities such as stores, schools, and workplaces that can easily be accessed by walking [6, 9]. One popular approach to measuring neighbourhood walkability is through Walk Score**®** [12]. This publically available metric is commonly used in real estate listings because of the positive connections between walkability and property values [12–14], and it has been employed by various scholars in studies on health and the built environment [9, 15–17].

Several studies have demonstrated a positive correlation between walkability and physical activity [6, 8, 9, 15–17], making the construction (either new or through retrofit) of walkable built environments a useful intervention to help address low levels of physical activity in the population [18, 19]. However, most published studies that aim to link walking behaviors with walkability are cross-sectional and ecological in nature, preventing researchers from being able to make causal inferences regarding the observed relationship [20]. Furthermore, cross-sectional, ecological designs in this area of research are vulnerable to selection bias: that is, individuals who are predisposed to be more physically active may choose to live in urban areas that offer more opportunities for regular physical activity, especially active transportation. Although some studies have attempted to statistically control for this bias [21, 22], it is widely acknowledged that longitudinal research on individuals and households is needed to fully control for potential selection bias [8, 9, 19, 21, 23].

A handful of studies have implemented longitudinal study designs to explore the relationship between walkability and physical activity [5, 18, 22, 24, 25], but the evidence is still fledgling. One study [25] collected longitudinal data that was focused on a limited sample size of African American women, making the findings less generalizable. Another quasi-experimental study from New Zealand [18] found increases in physical activity levels to be commensurate with investments in infrastructure and programming to support walking. The rest of the studies [5, 21, 22] all drew from the same dataset gathered from residents in Perth, Australia. To our knowledge, there are few, if any, longitudinal studies that examine changes in physical activity patterns after residential relocation to a more or less walkable environment, nor are there any studies in North America that draw from a more broad-based population.

Pilot studies [26], also known as pre-studies, are “intended to assess the safety of treatment or interventions; to assess recruitment potential; to assess the feasibility of international collaboration or coordination for multicentre trials; to evaluate surrogate marker data in diverse patient cohorts; to increase clinical experience with the study medication or intervention, and identify the optimal dose of treatments for the phase III trials”. Ultimately, they help to determine if investments in more substantial and powerful studies are warranted, and that is the over-arching purpose of the current study. Accordingly, our study was guided by three objectives. First, we sought to determine if it was possible to recruit and retain a longitudinal sample of people from households that were known to be moving residential location imminently, and if the recruits’ planned moves would entail enough change in the walkability of their residential environment to provide adequate counter-factuals for study. Second, we sought to determine whether perceptions of the neighbourhood and residents’ self-efficacy for walking changed from baseline to follow-up, and whether this change was associated with a change in neighbourhood walkability. And third, we sought to determine if we could measure pre- and post-move physical activity with a tolerable level of respondent burden and accuracy, and whether the direction of change was consistent with expectations. Specifically, we hypothesized that as participants moved residential location to neighbourhoods with built environment attributes that are more supportive of physical activity, their physical activity levels would increase. Similarly, if participants moved to neighbourhoods with less supportive built environments, we hypothesized that physical activity would decrease. Results from such a study would fill a significant gap in the current literature on urban form and physical activity.

## Methods

To meet our study objectives, we conducted a pre-post longitudinal study of changes in neighbourhood walkability and physical activity by employing a quasi-experimental approach to produce stronger evidence for a causal relationship between the built environment and physical activity levels. We recruited a convenience sample of participants who were identified as ‘imminent movers’ from publicly available real estate listings, and assessed neighbourhood walkability for both pre-move and post-move residences, as well as their neighbourhood perceptions, self-efficacy for walking, and physical activity levels before and after their move.

Data for the study was collected by researchers based in the city of Hamilton, Ontario. Eligible study participants were those living in a census metropolitan area (CMA), census division (CD), or census subdivision (CSD) in the province of Ontario, although the bulk of our participants were from the Greater Golden Horseshoe Area. Geographically, our eligibility criteria covered the entire urban and suburban population of Southern Ontario, while excluding rural areas as well as urban and suburban communities in Northern Ontario.

### Recruitment

The most expeditious approach to identifying imminent movers for a study such as this is to use residential real estate listings are that publicly available in Canada through the website www.realtor.ca. However, the Terms of Use for www.realtor.ca forbid scraping of the website or use by ‘institutions’ (as opposed to individuals, which is the intended use), without permission from the Canadian Real Estate Association (CREA). Unfortunately CREA declined our request to allow www.realtor.ca for participant recruitment for this study, so we resorted to an approach that was legal, albeit labour intensive and only produced a convenience sample. We used a variety of approaches to identify real estate advertisements, including real estate adverts in newspapers, ‘for sale’ signs visible in the research team’s daily routine activities. In addition, we searched for, and followed, Twitter and Facebook to build up lists of real estate agents operating in Southern Ontario to learn about new listings as they came available. We focused our recruitment efforts from March to November of 2014, which is typically the busiest period for real estate sales in this area of Canada.

From the addresses obtained by these methods, reverse lookups were performed using 411.ca and Canada411.ca to find the contact information (i.e., last name and phone numbers) of the property owners. These websites are created using landline telephone listings from large providers of home-based telecommunication services in Canada. These reverse lookups provided 75% coverage for telephone numbers among the properties we identified for sale, but the information wasn’t always current since Canada411.ca and 411.ca is estimated to be as much as 2 years old. If a property we identified had out-of-date names and telephone numbers, we still attempted to contact the current owner-occupier by letter without a named addressee.

Having identified a convenience sample of imminent movers for potential recruitment, we mailed targeted households letters using the address of the residence that they had listed for sale. A reply card and a stamped reply envelope were included in this letter for the individuals to mail back their desire to participate in the study. A space was also included to provide contact information. Participants were followed up with a phone call 2 weeks later to confirm their identity, and that they were indeed selling their house and planning to move residential location. Those who indicated their interest in participating were sent a letter explaining the survey procedures, and informing them of ethical considerations.

Overall, we attempted to contact 1421 potential movers by mail. Of these, we made contact with 537 potential participants while the remaining 884 we were unable to contact. Reasons for failing to contact potential participants included: phone numbers out of service (569); no response to mailing or follow-up calls (255); and letter returned undeliverable (50). Among those households with whom we did make contact, 143 were deemed ineligible (usually because they were no longer moving), 335 declined to participate and 59 agreed to participate. Among the 59 baseline participants, 19 were lost to attrition, 4 did not end up moving, and 1 moved to another unit in the same apartment building. The current paper reports the findings for the remaining 35 households for which we had complete pre- and post-move data for two different locations. At baseline and follow-up, all survey participants were issued a detailed letter of information about the study, and provided their written consent prior to participating. The study received ethics approval in April 2014 from McMaster University Research Ethics Board (protocol #2014–089).

### Data collection

Individuals who agreed to participate in the study were mailed a diary to track their movements for an average week, allowing us to compile a spatio-temporal inventory for each participant. They were also assigned a date to participate in a 45-min phone interview to capture demographic variables, perceptions of their neighbourhood, self-efficacy for walking and physical activity, activity levels from an average day from the completed activity diary, and other times and contexts in which they were active. The goal of the diary and interview was to establish the most extensive coverage of any physical activity done by participants in a typical day. Participants moved residential location at some time after our baseline interview, and roughly 1 year after baseline data collection (mean 355 days, range 297 to 439 days), we conducted a follow-up phone interview that asked the same questions as baseline. Participants also completed the same physical activity diary for their new residential location. While the length of time between selling and closing a home is highly variable, we estimate that most participants had been living in their new home for at least 6 months at the time that our follow-up survey was administered. Participants were compensated $40 for every phone interview they completed.

### Measurements and analysis

According to current literature [7, 8, 22, 23], time spent walking is impacted more by the built environment than total physical activity, and walking for the purpose of transportation (time walking for commute, time walking for errands) is affected more by the built environment than walking for recreational purposes [7, 8, 22]. As such, we measured time walking for utilitarian purposes (i.e., commuting, errands, walking child to school), as well as time spent engaged in job-related and recreational physical activities. Utilitarian-related, job-related, and recreational physical activity minutes were then converted to metabolic equivalents (METS) to facilitate comparisons from baseline to follow-up. To do this, activities reported by participants were classified as mild intensity, moderate intensity, and vigorous intensity and time spent doing each activity was multiplied by a coefficient to convert that activity to METS (Mild × 2.4, Moderate × 4.3, Vigorous × 6.5) [27]. All utilitarian-related walking times were automatically classified as moderate intensity.

In previous research, neighbourhood perceptions and self-efficacy have been found to be an important determinant of physical activity [28], and linked to the built environment [19]. Thus, we assessed perceptions of the neighbourhood for walking and self-efficacy for walking using a 7-point Likert scale, where 1 represents low perceived importance and low self-efficacy and 7 represents high perceived importance and high self-efficacy. Demographic variables that have been linked to physical activity and transport behaviour such as gender and level of education were also collected [29, 30].

Using participants’ self-reported postal codes, neighbourhood walkability was assessed using the publicly available online tool known as Walk Score**®** [12]. After inputting an address into the website’s search tool, Walk Score**®** assigns a numerical score from 0 to 100, with 0 corresponding to “car-dependent” and 100 to “walker’s paradise” [12]. The score is generated through an analysis of the walking distance to nearby amenities, which awards points based on proximity to those amenities from the origin address inputted into the tool. According to the Walk Score**®** website, “a decay function is used to give points to more distant amenities, with no points given after a 30 minute walk”, and the score also accounts for “population density and road metrics such as block length and intersection density”. While Walk Score**®** has been critiqued for oversimplifying the complex phenomenon of walkability and for failing to account for quality of amenities and the pedestrian experience [14, 31], validation studies have consistently found that Walk Score**®** closely corresponds to both objective and subjective measures of the built environment [32–34], and is thus an accurate metric for walkability. Indeed, its accuracy and ease-of-use made it an ideal walkability metric for this pilot study.

We created two walkability categories based on the Walk Score**®** values: low walkability (scores ranging from 0 to 59), and high walkability (60–100). These categories were used since Walk Scores**®** below 60 are generally considered to be not walkable [12, 33]. Once baseline and follow-up interviews were completed, participants were sorted into categories based on the Walk Score**®** of the different neighbourhoods they moved to. These ‘Walk Score**®** change groups’ were identified as Low-Low (L-L), Low-High (L-H), High-Low (H-L), and High-High (H-H). Given the small sample size and multiple analyses, we limit our description of the results to the directionality of change and the apparent relative magnitude.^1^

## Results

### Demographic characteristics and Neighbourhood walkability profiles

**Fig. 1:**
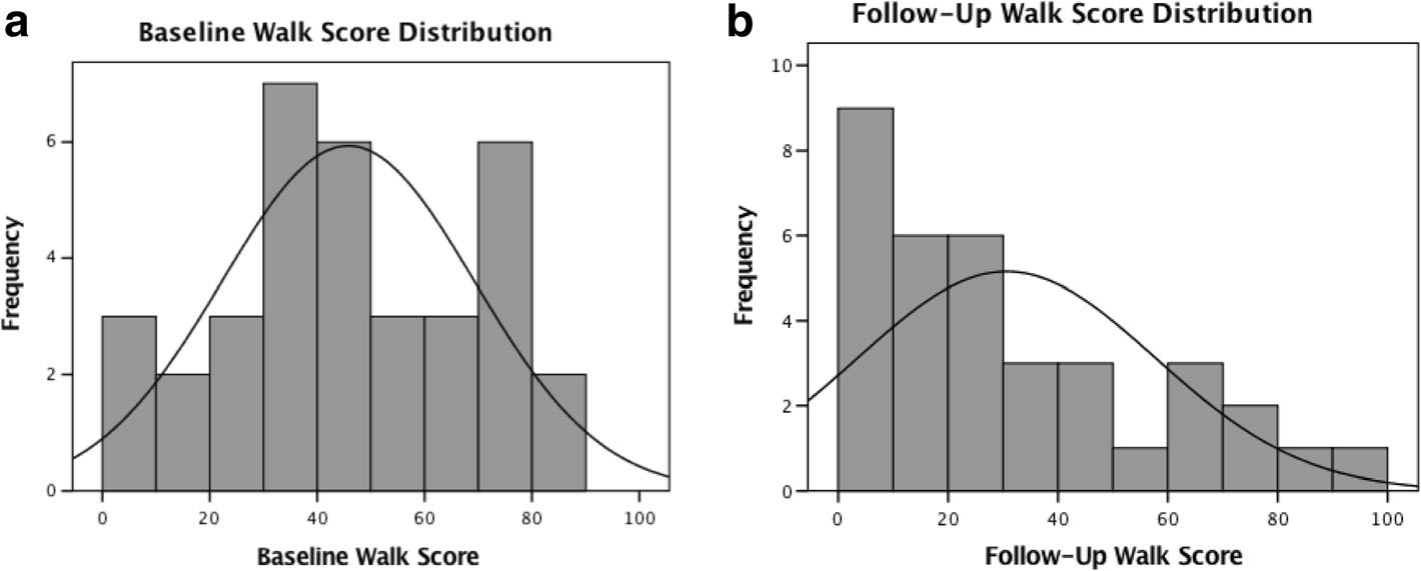
**a** and **b** Baseline and Follow-Up Distributions of the Walk Score® Values of Participants' Home Addresses

The majority of participants were female (71%) and had a college diploma or higher (80%), and the average age was 51.2 years (±15.2). Of the 35 participants, 19 were placed in the L-L group, 5 in the L-H group, 9 in the H-L group, and 2 in the H-H group, although no analyses were done on this latter group alone. Figure 1a and b illustrate the distribution of Walk Scores**®** at baseline and follow-up. When examining the sample as a whole, we observed a decrease in the Walk Score**®** from baseline to follow-up, from an average of 45.8 to 30.6.

### Baseline vs. follow-up Neighbourhood characteristics

**Fig. 2:**
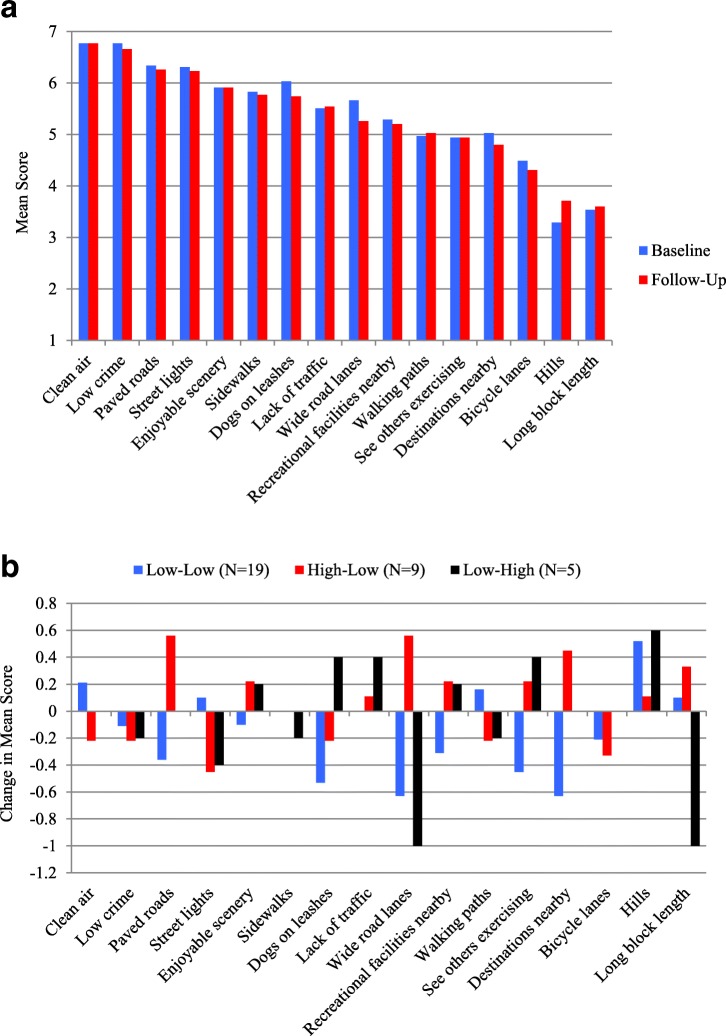
**a** Mean Level of Importance of Various Neighbourhood Characteristics for Walking, at Baseline and Follow-up, for the Entire Sample (*n* = 35). **b** Change in Mean Level of Importance of Neighbourhood Characteristics for Walking, from Baseline to Follow-up, for Each Walk Score® Change Group

A series of questions were posed to participants, at baseline and follow-up, regarding the importance (on a scale of 1 (low) to 7 (high)) of various characteristics on walking in their neighbourhood. Figure 2a displays the mean scores, at baseline and follow-up, for the entire sample in descending order of scores at follow-up. The neighbourhood characteristics that were considered most important for walking at both baseline and follow-up were *clean air*, *low crime, street lights*, and *paved roads.* Meanwhile, neighbourhood features considered to be least important for walking were *block length* and *presence of hills*. Similar patterns in terms of overall levels of importance were observed among the Walk Score**®** change groups (results not shown); the main point of differentiation came from the L-H group which placed high importance at baseline and follow-up on the presence of *sidewalks* and notably less importance on the presence of *paved roads*.

Figure 2b displays the differences, from baseline to follow-up, in the mean level of importance of the neighbourhood characteristics for walking for each Walk Score**®** change group. This figure illustrates two key points: 1) how relatively stable these neighbourhood perceptions were over time, such that the magnitude of changes in mean scores never exceeded ±1; and 2) the variations between the three groups in terms of which neighbourhood characteristics matter more or less after their move. For the H-L group, *paved roads* (+ 0.56) and *destinations nearby* (+ 0.45) were more important for walking at follow-up. For the L-L group, *dogs on leashes* (− 0.53) and *destinations nearby* (− 0.63) were less important at follow-up. For the H-H group, *long block length* (− 1.00) was less important at follow-up. The greatest divergence in perceptions was observed for *wide road lanes*; this characteristic increased in importance for the H-L group (+ 0.56), and decreased in importance for both the L-L (− 0.63) and the H-H group (− 1.0), at follow-up.

### Baseline vs. follow-up self-efficacy for walking

**Fig. 3:**
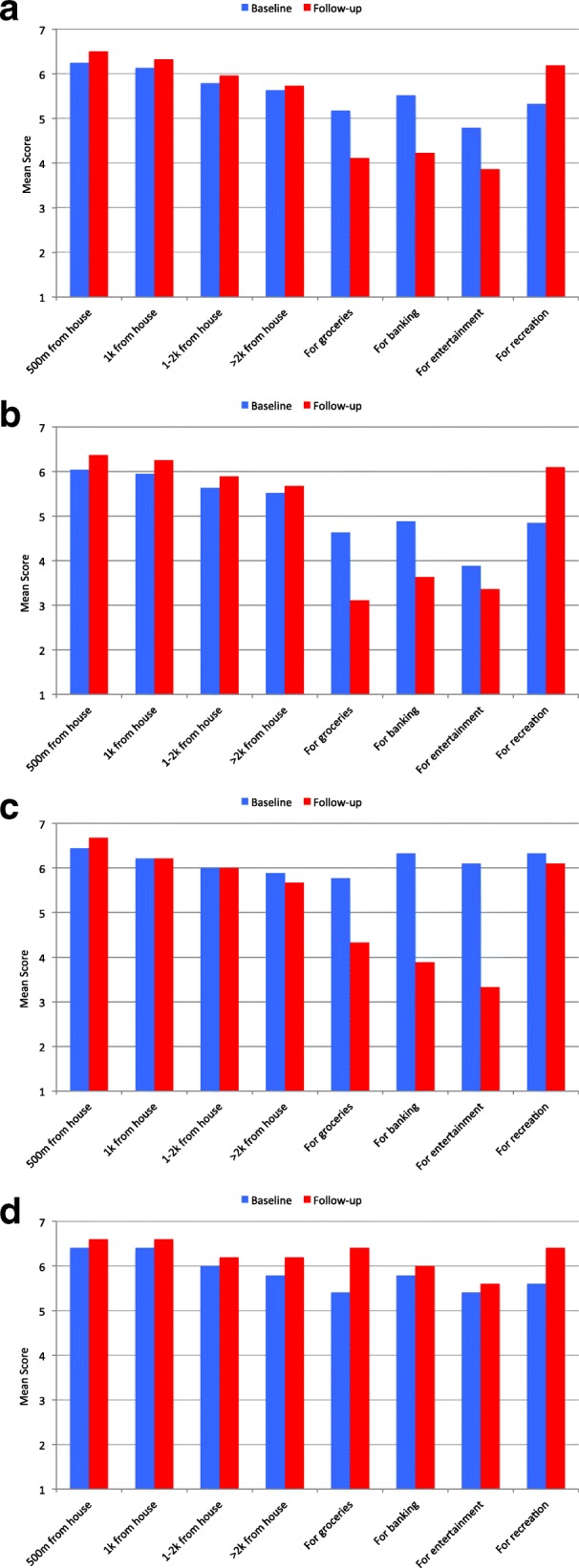
**a** Mean Level of Self-Efficacy for Walking, at Baseline and Follow-up, for the Entire Sample (n = 35). **b** Mean Level of Self-Efficacy for Walking, at Baseline and Follow-up, for the Low-Low Walk Score® Change Group (*n* = 19). **c** Mean Level of Self-Efficacy for Walking, at Baseline and Follow-up, for the High-Low Walk Score® Change Group (*n* = 9). **d** Mean Level of Self-Efficacy for Walking, at Baseline and Follow-up, for the Low-High Walk Score® Change Group (*n* = 5)

Another series of questions regarding their self-efficacy for walking for various distances and purposes (on a scale of 1 (low) to 7 (high)) were posed to participants, at both baseline and follow-up. Figure 3a-d display the mean scores, at baseline and follow-up, for the entire sample and the three Walk Score**®** change groups. For the entire sample and for each Walk Score**®** change group, as distance increased, perceived self-efficacy for walking decreased at both baseline and follow-up. For the entire sample (Fig. 3a) and for the L-L group (Fig. 3b), self-efficacy for walking was lower, at both baseline and follow-up, for walking to destinations (i.e., *groceries, banking, entertainment*) as compared to walking for *recreation*. It is noteworthy, however, that self-efficacy for walking to destinations decreased substantially from baseline to follow-up for the H-L group (Fig. 3c), and that self-efficacy for walking was consistently high (i.e., score > 5) at baseline and follow-up for every distance and purpose for the L-H group (Fig. 3d). Overall, it is evident that for the entire sample, self-efficacy for walking increased from baseline to follow-up for the *500 m from home* distance and *for recreation*, and decreased for walking *for groceries*, *for banking*, and *for entertainment*.

### Baseline vs. follow-up physical activity levels

**Fig. 4:**
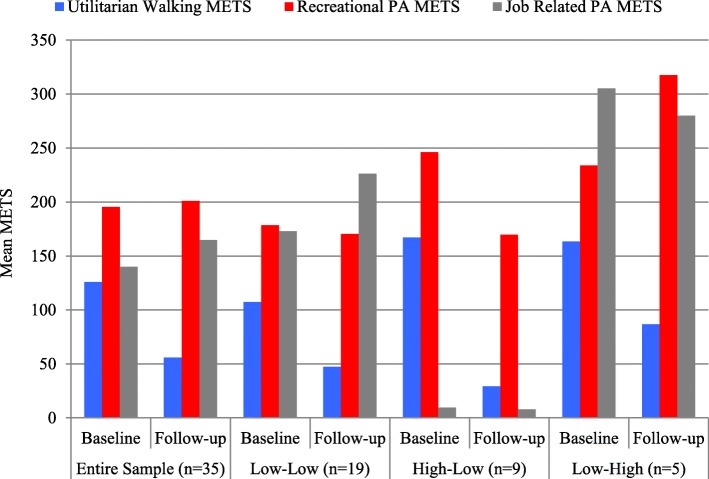
Mean METS for Utilitarian Walking, Recreational Physical Activity, and Job-Related Physical Activity, at Baseline and Follow-up, for the Entire Sample and the Walk Score® Change Groups

Figure 4 summarizes the mean METS at baseline and follow-up generated from utilitarian walking, recreational PA, and job-related PA for the entire sample and the three Walk Score**®** change groups. For the entire sample, mean METS were highest, at baseline and follow-up, for recreational PA. Meanwhile, utilitarian walking METS decreased from baseline to follow-up for the entire sample and all three Walk Score**®** change groups. The L-H group had the highest job-related PA, at baseline and follow-up, of all three Walk Score**®** change groups, and it was the only group that had an increase in recreational PA METS from baseline to follow-up.

## Discussion

Despite the need, there are few studies of the effect of the built environment on physical activity that involve a change in individuals’ exposure to a walkable neighbourhood. In this longitudinal pilot study, we sought to determine: i) whether we could recruit and retain a longitudinal sample of people from households that were known to be moving residential location imminently, and if the recruits’ planned moves would entail enough change in the walkability of their residential environment to provide adequate counter-factuals for study; ii) whether perceptions of the neighbourhood and residents’ self-efficacy for walking changed from baseline to follow-up, and whether this change was associated with a change in neighbourhood walkability; iii) whether we could measure pre- and post-move physical activity with a tolerable level of respondent burden and accuracy, and whether the direction of change was consistent with expectations.

Our attempt to recruit a sample of imminent movers from real estate listings revealed some of the challenges in conducting such a study. Prior to commencing the study, we formulated two plausible, but contradictory hypotheses about the willingness of imminent movers (households trying to sell their residence) to participate in a study on physical activity. On the one hand, we thought that people who were in the midst of finding a new place to live would be ‘primed’ to participate in a study that asked questions about neighbourhood quality, given their personal immersion in the topic while they searched for a new home (or just completed such a search). On the other hand, we thought that given the stressors associated with buying, selling a residence, and moving, that people would be too busy and/or stressed to participate. Although we don’t have comprehensive direct evidence of the role of stress, it does seem that the latter is true and imminent movers are disinclined to participate in research, likely due to time stress and possibly other reasons. In short, and despite our best efforts, it appears to be very difficult and costly to recruit even a convenience sample of imminent movers that is sufficiently large to study. To overcome these challenges, future studies could consider partnering with real estate boards and rental housing agencies to enable researchers to mine all available real estate and rental listings for recruitment purposes, and/or partner with individual real estate and rental agents who can recruit willing participants on the researchers’ behalf.

Among the small number of people who did participate in our study, we also sought to determine if the participants’ residential moves would entail enough change in the walkability of their residential environment to provide adequate counter-factuals for study. With a small sample, we were only able to assess this change in exposure in a binary fashion, and classified peoples’ moves as High-High, High-Low, Low-High and Low-Low. Ideally, it would be possible to classify people according to quartiles or quintiles of walkability at both baseline and follow-up, but of course that also demands a larger sample. Within the constraints of our sample size and binary walkability classification, we were pleasantly surprised at the number of participants who moved from Low to High walkability areas (5 of 35), but we also noted the number who moved from High to Low (9 of 35). This is understandable given the predominantly suburban urban form in cities in the metropolitan areas of province of Ontario, and is reinforced by the fact that more than half of participants moved from one Low walkability area to another Low walkability area (19 of 35). This signals an important structural challenge to conducting studies such as this one – relatively few people who move residence, move from low walkability areas to high walkability areas, perhaps because they are under-supplied [35].

Some interesting findings emerged with regards to our second study objective. Specifically, residents’ perceptions of their neighbourhood were consistent from baseline to follow-up for the entire sample, and for the three Walk Score**®** change groups, despite the fact that nearly half the sample moved to a neighbourhood with a contrasting walkability profile (i.e., 9 moved from high to low, and 5 from low to high). At baseline and follow-up, the neighbourhood characteristics that were deemed most important for walking among the entire sample were *clean air*, *low crime*, *paved roads*, and *street lights*, which all generated mean scores of greater than 6 on our 7 point scale. Meanwhile, *presence of hills* and *long block lengths* were viewed as the least important characteristics, generating mean scores of less than 4 out of 7. In terms of the Walk Score**®** change groups, our only major finding from this analysis was that the L-H group rated the presence of sidewalks more highly than the other two groups. These findings suggest that, regardless of the built form of a neighbourhood, there is some universality in terms of the neighbourhood characteristics that matter most and least for encouraging residents to get out and walk.

We detected interesting findings with respect to self-efficacy for walking, in terms of change from baseline to follow-up, and in terms of differences between the three Walk Score**®** change groups. For the entire sample, self-efficacy for walking was highest (mean score above 6 on our 7 point scale) at baseline and follow-up for *500 m and 1 km from home*, and at follow-up *for recreation*. For all measures, self-efficacy for walking increased for the L-H group from baseline to follow-up. By comparison, self-efficacy for walking consistently declined or stayed the same for the H-L group, and declined for some measures and increased for others amongst the L-L group. This reinforces the notion that built environment characteristics may affect self-efficacy for walking, and despite the crude distinction between high and low walkability, the results also suggest that when people move to a different level of walkability, the measurement of self-efficacy for walking detects something consistent with expectations. It is also important to note however that, unlike the L-L and H-L groups, self-efficacy for walking scores were consistently above 5 at baseline and follow-up for the L-H group, suggesting that these movers were seeking a new neighbourhood that would support and enable their existing capacities for walking.

In terms of our third objective, we found that among the participants we recruited, the data collection procedures (travel diary, conversion to physical activity intensity levels and METS; survey on determinants of activity) were successful. We achieved our goal of limiting the baseline and follow-up interviews to 45 min each (39 min on average), which is the key indicator of acceptable burden. The data were mostly of high quality and we did not lose any cases due to poor quality data, which is especially heartening since participants completed travel diaries on their own for a whole week. In terms of our PA related findings from baseline to follow-up, we observed a large decline in utilitarian PA (from 125 to 56 Mean METS), a modest increase in job-related PA (from 140 to 165), and a marginal increase in recreational PA (from 195 to 201). Surprisingly, we not only observed a decline in utilitarian PA for the L-H group (from 163 to 87), but this was also the only group that exhibited an increase in recreational PA (234 to 318), from baseline to follow-up. These latter findings ran counter to our expectations, but may highlight the important of close proximity to recreational amenities in high-density areas for physical activity, which is something that is less frequently emphasized than utilitarian walking.

The only previous study that was successful in recruiting imminent movers involved partnering with a government agency to contact individuals who planned on moving to various state-approved housing developments [5]. Such a strategy, however, necessarily limits the generalizability of the findings to a narrow cross-section of the population. We know of no other studies that have successfully recruited and followed a group of imminent movers that represent a broad cross-section of the population and studied the effect of changes in built environment on physical activity. A study in Ontario, Canada [36] used survey data from a national survey linked to administrative health care records and found that among people who changed address in the administrative databases, moving to a highly walkable neighborhood reduced the risk of incident hypertension; but such methods are limited to health outcomes that can be assessed using administrative health care utilization data. The use of such outcomes instead of physical activity may introduce new, unmeasured confounders, since the causal chain between built environment and physical activity is more direct than the causal chain between built environment and hypertension, for instance.

One of the other challenges associated with conducting a study such as this one is that that built environment constrains the options available to people moving. Gordon and Shirokoff [37] examined the distribution of urban form across Canada, and found that 80% of Canadians are living in “suburban” communities and another 10% are living in “exurban” communities, both of which are characterized by having automobiles as the primary form of transportation and low walkability scores. Meanwhile, only 10% of Canadians are living in “urban” (i.e., walkable) communities, where public transit or walking are the primary methods of transportation. Thus, the relatively small proportion of properties that are located within highly walkable communities in North American cities means that large sample sizes are needed to achieve enough movement in the sample. Recent data allows for a similar observation from a slightly different perspective: that mixed-use neighbourhoods are significantly under-supplied in some Canadian cities (and very expensive) [35].

Finally, it is important to note that our substantive findings must be interpreted with caution for a few reasons. First, the sample size limited our analyses to directionality and apparent relative magnitude of change only. We hope that future studies will overcome the recruitment challenges that we encountered to enable inferential analyses based on changes in walkability. Second, our built environment metric relied exclusively on Walk Score®, which has been criticized for oversimplifying the complexities of the built environment while failing to account for the pedestrian experience [14, 31]. Despite these critiques, Walk Score® has been validated against both subjective and objective assessments of walkability [34], and its ease of use made it an ideal metric for this pilot study. Larger scale quasi-experimental studies should consider supplementing the use of Walk Score® with additional indicators (e.g., sidewalk presence and quality, traffic speed and volume, types of amenities, etc.) that offer more nuanced details on the conditions of the built environment. Third, the elapsed time between people’s baseline and follow-up data collection is quite variable, partly because of variation in the time between baseline and the sale of the participants’ houses, and then again because of variability in the period between sale of the house and move date for each individual. We conducted ‘check-ins’ with participants 6 months after their baseline interview to see if they had moved and then followed up accordingly based on what they told us. We are confident that the majority of participants had been living in their new home for at least 6 months at the time of their follow-up survey, but we lack the sample size to determine if variability in this or other elapsed time periods makes a difference to the outcomes. Any modeling in future studies should account for variable time between baseline and follow-up and intervention (moving) and follow-up data collection. Finally, there is a tremendous variety of confounding variables that exist when assessing the routines of people throughout their life. Physical activity, the primary outcome variable of this study, is just a small portion of what people do every day. Daily routines, unforeseen occurrences, changes in household composition (e.g., family dissolution), stage of life-cycle, and changes in life experience can all have impacts on one’s living routine, especially physical activity.

## Conclusions

The foregoing suggests the need for additional research on the impact of changes in residential built environment and physical activity to inform policy in this area. We have identified a number of challenges in recruiting a sample of imminent movers of sufficient size to power studies that can meet the research needs. There is some promise in studies that use linked survey and health care administrative data, but these cannot directly measure physical activity changes or control for the confounders that are inevitable with longer causal chains. Future studies of this nature might have better success with recruitment if the researchers are able to strike partnerships with real estate boards and rental agencies. It is imperative, however, that public health agencies and scholarly funding bodies prioritize resources for longitudinal studies investigating the impact of built environment changes on physical activity.

## Abbreviations

H-H: High-High
H-L: High-Low
L-H: Low-High
L-L: Low-Low

## Footnotes

1 Descriptive statistics were not calculated for the H-H Walk Score**®** change group because of the *N* = 2 sample size.
